# Influence of Guar Gum, Arabic Gum, and Stevia on the Physicochemical, Rheological, and Microbial Properties of Kiwi‐Based Sauces

**DOI:** 10.1002/fsn3.70174

**Published:** 2025-04-14

**Authors:** Seyede Golsa Mousavi, Leyla Alizadeh, Mostafa Karami

**Affiliations:** ^1^ Department of Food Quality Control and Hygiene, Science and Research Branch Islamic Azad University Tehran Iran; ^2^ Department of Food Science and Technology, Faculty of Food Industry Bu‐Ali Sina University Hamedan Iran

**Keywords:** guar gum, kiwi sauce, microbial properties, physicochemical properties, stevia

## Abstract

This work aimed to evaluate the effectiveness of incorporating varying levels of guar gum and Arabic gum (0.2%, 0.3%, and 0.4%) in kiwi sauce formulations, using stevia as a sugar substitute in the formulation. Herein, an endeavor has been made to detail the physiochemical properties, rheological behavior, and sensory attributes of the samples of sauce. Acidity was significantly affected by sugar type, except in unpasteurized kiwi sauce with stevia, while gum concentration had no significant effect on acidity (*p* > 0.05). The moisture content was increased with the addition of guar gum (*p* < 0.05) and dry matter content increased with higher Arabic gum concentrations (*p* < 0.05). The stevia‐sweetened sauce had the lowest dry matter content. Solid content increased linearly with increasing guar and Arabic gum concentrations. There was no significant difference among the color attributes of the samples (*p* > 0.05). Microbiological analysis revealed significant differences in mold and yeast growth among treatments (*p* < 0.05). Sensory evaluation showed that the sauce with 0.4% guar gum received the highest hedonic score. While the viscosity of all samples decreased during storage, the sauce with 0.4% guar gum retained the highest viscosity. In conclusion, this research demonstrates that incorporating hydrocolloids and stevia can significantly alter the physicochemical and sensory characteristics of kiwi sauce, improving its stability and consumer acceptability.

## Graphical abstract

This study assessed the impact of guar gum and Arabic gum (0.2%, 0.3%, 0.4%) with stevia as a natural sweetener on kiwi sauce. The best formulation was the kiwi sauce with 0.4% guar gum. Key findings include: The moisture content was increased by guar gum addition, and dry matter content increased with higher Arabic gum concentrations. Stevia‐sweetened sauce had the lowest dry matter. Highest sensory score belonged to sauce with 0.4% guar gum with enhanced viscosity and improved stability.

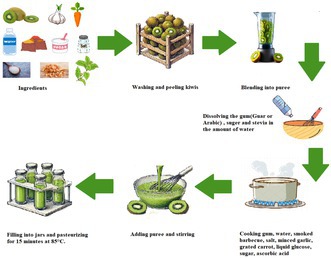

## 1 Introduction

Sauces form an integral part of many consumers' daily lives and constitute a significant part of human nutrition. One of the primary advantages and roles of sauces is in enhancing or changing the taste and odor of different food products (da Silva et al. 2021; Rahman et al. 2024). The attention, however, has been more toward the nutritional properties of the sauces, especially the formulation of bioactive sauces rich in bioactive compounds that are beneficial and originated from fruits and vegetables, which has become a serious concern now (Rahman et al. 2024). Now, among the different types of sauces, fruit‐based sauces have gained noteworthy attention in the food industry for their functionality and health‐promoting activities. This increased interest in the inclusion of fruits in sauce formulations relates directly to the increased public awareness concerning the diet and health link. Besides that, the usage of fruits in sauces and other processed foods enhances the organoleptic characteristics of some dishes. Kiwi, apple, apricot, pomegranate, among others, have been used in making fruit sauces (Mahrus et al. 2020).

Kiwi is a very nutrient‐rich fruit with low caloric value. This is a good source of many essential micronutrients, like calcium, iron, and potassium. Kiwis have abundant amounts of vitamin C and fiber coupled with the mineral's such as potassium and magnesium, along with chlorophyll and carotenoids. The high dietary fiber content of kiwi, about 10% of the daily recommendation, in conjunction with its low energy density and antioxidant properties, helps qualify it as one of the healthiest fruits available. According to research, the consumption of kiwis is related to a lot of health benefits associated with their rich content in phenolic compounds, such as their anticancer effects and antioxidant benefits, reductions in cholesterol level, and enhancement of gut health and functionality (Sanz et al. 2021). However, kiwi is a relatively short‐lived fruit and really prone to big waste generation. Reports indicate that wastes originating from kiwi production and processing constitute about 30% of the total yield. Such wastes, ranging from the asymmetrical shape of the fruit itself to by‐products of processing, such as the pulp resulting from kiwi juicing, have been documented (Sharma et al. 2016). In light of these observations, one strategy for reducing losses/wastes while at the same time extending kiwi's fresh life is to transform kiwi into value‐added products, among them kiwi sauce. Nevertheless, the generation of fruit sauces with several attractive properties is a subtle process since it is highly influenced by the conditions of processing and formulation that affect the quality and consumer acceptance of the final product (Levent and Alpaslan 2018). Cervera‐Chiner et al. (2021) addressed the replacement of traditional sugars with non‐centrifugal sugars in the formulation of functional jams from strawberries and kiwis. Work was focused on the substantiation of how this substitution affects physicochemical characteristics, antioxidant capacities, and sensory attributes in the case of jams. Satpal et al. (2021) present a comprehensive review on *Actinidia deliciosa* , commonly referred to as kiwi fruit, on its nutritional composition, health benefits, traditional uses, and commercialization. This study describes the high level of nutrition found in kiwi: it contains a long list of nutrients, starting from vitamins and minerals to antioxidants that have been associated with different positive impacts on health, such as improved digestion, enhanced immune function, and cardiovascular health. The Kiwifruit sugar coefficient unique identification using near‐infrared spectroscopy and deep learning is a study by Liu et al. (2023). It aims to come up with a non‐destructive and quick guideline to determine sugar levels accurately, which is critical for the fruit industry. That, in fact, from the experiments, they showed that the use of the proposed deep learning model is able to significantly increase the accuracy of sugar content classification as opposed to traditional methods. This might mitigate the efficiency of kiwi fruit processing and quality assessment and correspond to its commercial applications. Another challenge in producing fruit sauces is their relatively thin consistency due to the lack of physical stability and the difficulty in maintaining a uniform product during storage (Ahouagi et al. 2021). To address this issue, hydrocolloids are commonly used to suspend particles in the product. In this regard, Pirsa and Hafezi (2023) reviewed the structure, methods of preparation, and uses of hydrocolloids in the food industry. It deals with their functioning as thickening, gelling, and stabilizing agents, enumerating upon their functionality toward the betterment of food texture and uplifting its quality. (Alam et al. 2023) examine the use of various hydrocolloids to enhance the techno‐functional, rheological, and nutritional properties of food fillings. The study explores how different hydrocolloids can modify texture, stability, and nutritional content. It highlights the potential of hydrocolloids to improve the quality and functionality of food products. Hydrocolloids such as Arabic gum, starch, xanthan, and guar gum are widely used in the sauce industry (Das and Chakraborty 2016). Moreover, the increasing public awareness of proper nutrition, which can positively impact people's health, has led to a greater preference for products with lower levels of fat, salt, and sugar. In the development of low‐sugar foods, a few sweeteners are used as alternatives to sugar. One prominent natural sweetener is stevia (Chitgar and Mansouri‐Pour 2017). The commercialization of its derivatives has been in the mainstream market of various food, beverage, and pharmaceutical industries globally with the launch of commercialized products, in the attempt to develop low‐sugar foods (Abbas Momtazi‐Borojeni et al. 2017). Given the increasing global demand for sauces and healthy foods, the aim of this study was to produce kiwi fruit sauce and evaluate its physicochemical, physical, chemical, and microbial stability to ultimately produce a product that can be marketed. Nemati and Hesarinejad (2024) investigate the application of hydrocolloids derived from *Echinops setifer* in mayonnaise‐type sauces. The study focuses on how these hydrocolloids can improve the texture and stability of sauces. It demonstrates the potential of *Echinops setifer* hydrocolloids as an effective alternative in food formulations.

Despite extensive research on the use of hydrocolloids in various food products, there is limited information on their specific effects in fruit‐based sauces, particularly those using alternative sweeteners like stevia. Most studies have focused on the general application of hydrocolloids for improving texture and stability in different food matrices or on the nutritional benefits of fruits like kiwi. However, the unique combination of guar gum and Arabic gum with stevia in kiwi sauce formulations has not been thoroughly investigated. This study addresses this research gap by evaluating the effects of these hydrocolloids and a natural sweetener on the physicochemical, rheological, and sensory properties of kiwi sauce. By exploring these parameters, our work provides new insights into the potential for creating healthier, more stable, and appealing fruit‐based sauces, contributing to the advancement of functional food products in the market.

## 2 Materials and Method

Kiwis were procured from the local market in Hamadan. Guar gum and Arabic gum were sourced from Sahar Company (Hamadan, Iran). The culture media and other chemicals used were manufactured by Merck (Darmstadt, Germany).

### 2.1 Preparation of Samples

The fresh kiwis underwent a thorough washing process with water. Subsequently, the skins of the fruits were removed, and the flesh was blended into a puree using a kitchen blender. When deemed necessary, the purees were subjected to heating to inactivate enzymes and facilitate pasteurization. Ultimately, the resulting puree was stored in sterilized glass containers within a refrigerator until required (Levent and Alpaslan 2018). For the preparation of the sauces, the formulation detailed in the accompanying table was employed. Initially, all components of the sauce, with the exception of the puree, were combined and pasteurized at a temperature of 85°C for a duration of 15 min. Following this, the mixture was cooled to 40°C, at which point a predetermined quantity of puree was incorporated into the pasteurized solution and mixed thoroughly. The optimization of three parameters, that is, concentration (0.2%–0.4% w/w), type of stabilizer (Arabic gum and guar gum), and stevia (0%–3%), was conducted using a one‐factor‐at‐a‐time methodology, while the other two parameters remained constant. Additional factors examined in this research included the decision to heat or not heat the puree, the specific heating conditions of the puree (including time and temperature), and the type of sweetener utilized (sugar versus stevia), which were assessed independently. Table 1 lists the ingredients used in kiwi sauce formulation.

**TABLE 1:** Base ingredients utilized in the formulation of kiwi sauce.

Ingredient	Percent by weight	Ingredient	Percent by weight
Grated carrot	1.5	Liquid glucose	7
Salt	0.1	Garlic	0.7
Smoked barbecue	0.5	Ascorbic acid	0.10
Water	20	Stabilizer	0.2–0.4
Kiwi puree	60–70	Sugar	0–3
Stevia	0–3		

Finally, the initial sauce samples were stored for 2 weeks to evaluate stability and overall acceptance. The optimal sample was then selected for further testing. The base ingredient composition for kiwi‐based sauce includes kiwi puree, sugar, lemon juice, and cornstarch are shown in Table 2.

**TABLE 2:** The basic composition of ingredients used in the formulation of sauce based on kiwi.

Compositions									
Ingredient	T1	T2	T3	T4	T5	T6	T7	T8	T9
Kiwi puree	60–70	60–70	60–70	60–70	60–70	60–70	60–70	60–70	60–70
Water	20	20	20	20	20	20	20	20	20
Salt	0.1	0.1	0.1	0.1	0.1	0.1	0.1	0.1	0.1
Smoked barbecue	0.5	0.5	0.5	0.5	0.5	0.5	0.5	0.5	0.5
Liquid glucose	7	7	7	7	7	7	7	7	7
Stevia	0	0	0	0	0	0	0.176	0	0.176
Sugar	3	3	3	3	3	3	0	3	0
Gum	0.2	0.3	0.4	0.2	0.3	0.4	0.4	0.4	0.4
Grated garlic	0.7	0.7	0.7	0.7	0.7	0.7	0.7	0.7	0.7
Ascorbic acid	0.1	0.1	0.1	0.1	0.1	0.1	0.1	0.1	0.1
Grated carrot	1.5	1.5	1.5	1.5	1.5	1.5	1.5	1.5	1.5

### 2.2 Physiochemical Tests

To conduct physicochemical analyses, the Brix of the samples was initially measured using a hand‐held refractometer at 25°C to determine the total soluble solids content. (Cherif et al. 2011) The dry matter content of the sauce samples was also measured by the gravimetric method after being heated in an oven at 102°C for 6 h. To measure pH, 5 g of the sauce sample was diluted in 50 mL of distilled water, and then the pH of the samples was determined using a pH meter.

In the titratable acidity test, the aforementioned diluted solution was used, and titration was performed until a pH = 8.1 was reached using a 0.1 normal sodium hydroxide solution (Sadler and Murphy 2010). The acidity of the samples was calculated based on citric acid using the following equation.

(1) %Acidity=0.1×F×V×MeqM×100

where V, F, $M_{\mathrm{eq}}$, and M denote volume of sodium hydroxide solution (mL), standardization factor of the sodium hydroxide solution, milliequivalents of citric acid, and initial weight of the sauce sample in solution, respectively.

To evaluate the degree of syneresis and phase separation, 5 g of the sample was placed in a 10‐ml falcon tube, and after centrifugation at 3000 rpm for 20 min, the separated aqueous phase was weighed. The following formula was used to calculate the amount of water:

(2) %Synersis=M1M×100

For the analysis of color properties, the sauce samples were evaluated on the first day and after 30 days of storage. After homogenization, the samples were placed on a plate designed for the Hunter Lab instrument, and the L*, a*, and b* parameters were recorded for each sample (Sadler and Murphy 2010).

In the study of rheological properties, the apparent viscosity and flow behavior of the samples were measured using a plate rheometer at various shear rates (1–100 s^−1^), and the data was fitted to the Power law model (Rao et al. 1986; Sadler and Murphy 2010; da Silva et al. 2021). This model describes the relationship between shear stress and shear rate using the consistency index and flow behavior index.

(3) $$\sigma =K\gamma^{n}$$

where *σ* is the shear stress (Pa), γ is the shear rate (s^−1^), K is the consistency index (Pa.s^n^), and n is the flow behavior index.

### 2.3 Microbial Test

To evaluate the microbial properties of the sauce samples, a mold and yeast count test was conducted after 1 month of storage to assess microbial growth over time (da Silva et al. 2021). To evaluate the sensory properties of the samples, a 5‐point hedonic scale was used. In this test, 15 g of each sample in a sealed plastic container was presented to 15 semi‐trained panelists, and parameters such as appearance, color, aroma, consistency and texture, taste, and overall acceptance were evaluated. In this evaluation, a score of 1 indicated the lowest acceptance and a score of 5 indicated the highest acceptance for each parameter (da Silva et al. 2021). Statistical analysis of the data was performed using a one‐factor repeated measures design. The obtained results were first subjected to a one‐way analysis of variance, and Duncan's multiple range test at the 95% significance level was used to compare the means and determine significant differences between treatments. Statistical analysis of the data was performed using SPSS version 20 software.

### 2.4 Statistical Analysis of Data

All analyses were performed using SPSS software version 21. The one‐way ANOVA was applied to compare the parametric features of different samples, and the significance of mean differences was determined (at the level of *p* < 0.05) by the Duncan's multiple‐range post hoc test. Experiments were conducted in triplicate, and results were reported as mean ± standard deviation.

## 3 Results

In present study, nine treatments have been used. The parameters including pH, acidity, moisture, dry matter, soluble solids in water, syneresis, color parameter, and mold and yeast growth, as well as rheological parameters of each treatment have been investigated. Table 3 lists the treatments examined in this study.

**TABLE 3:** The treatments used in this study.

No.	Treatment	Formulation
1	T1	Kiwi sauce contains 0.2% guar
2	T2	Kiwi sauce contains 0.3% guar
3	T3	Kiwi sauce contains 0.4% guar
4	T4	Kiwi sauce contains 0.2% Arabic gum
5	T5	Kiwi sauce contains 0.3% Arabic gum
6	T6	Kiwi sauce contains 0.4% Arabic gum
7	T7	Kiwi sauce containing stevia
8	T8	Unpasteurized kiwi sauce containing sugar
9	T9	Unpasteurized kiwi sauce containing stevia

### 3.1 pH

**FIGURE 1:**
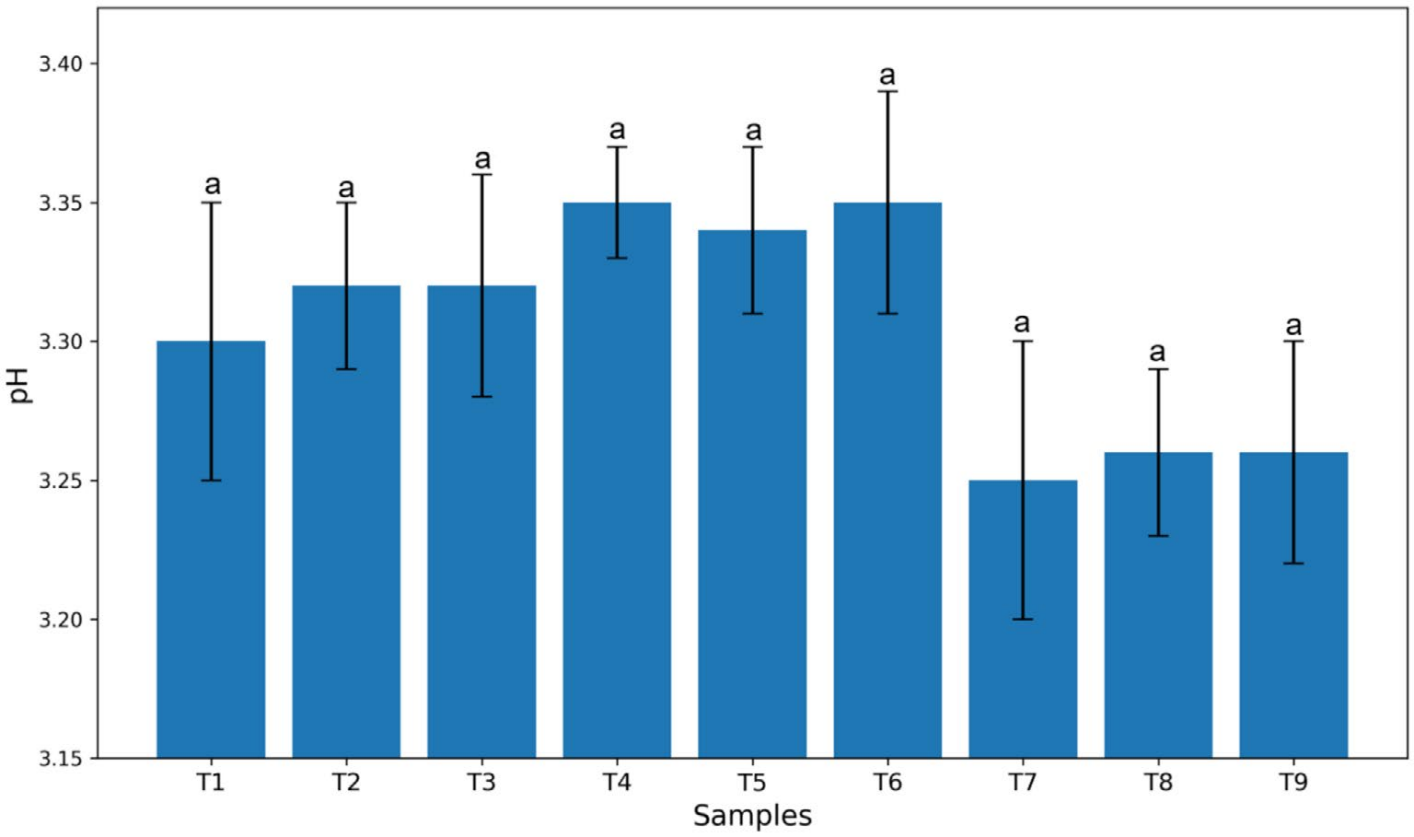
Effect of guar and Arabic gum levels on the average pH of kiwi sauce (*n* = 3), Bars represent mean ± SD. Different letters on the bars indicate significant differences among samples (*p* < 0.05).

The effect of different levels of Arabic and guar gums on the average pH is presented in Figure 1. The results showed that there was no significant difference in pH among all treatments (*p* > 0.05), and no significant change in pH was observed. As can be seen, the treatment containing only stevia had a lower pH compared to the other treatments (pH = 3.25).

### 3.2 Acidity

**FIGURE 2:**
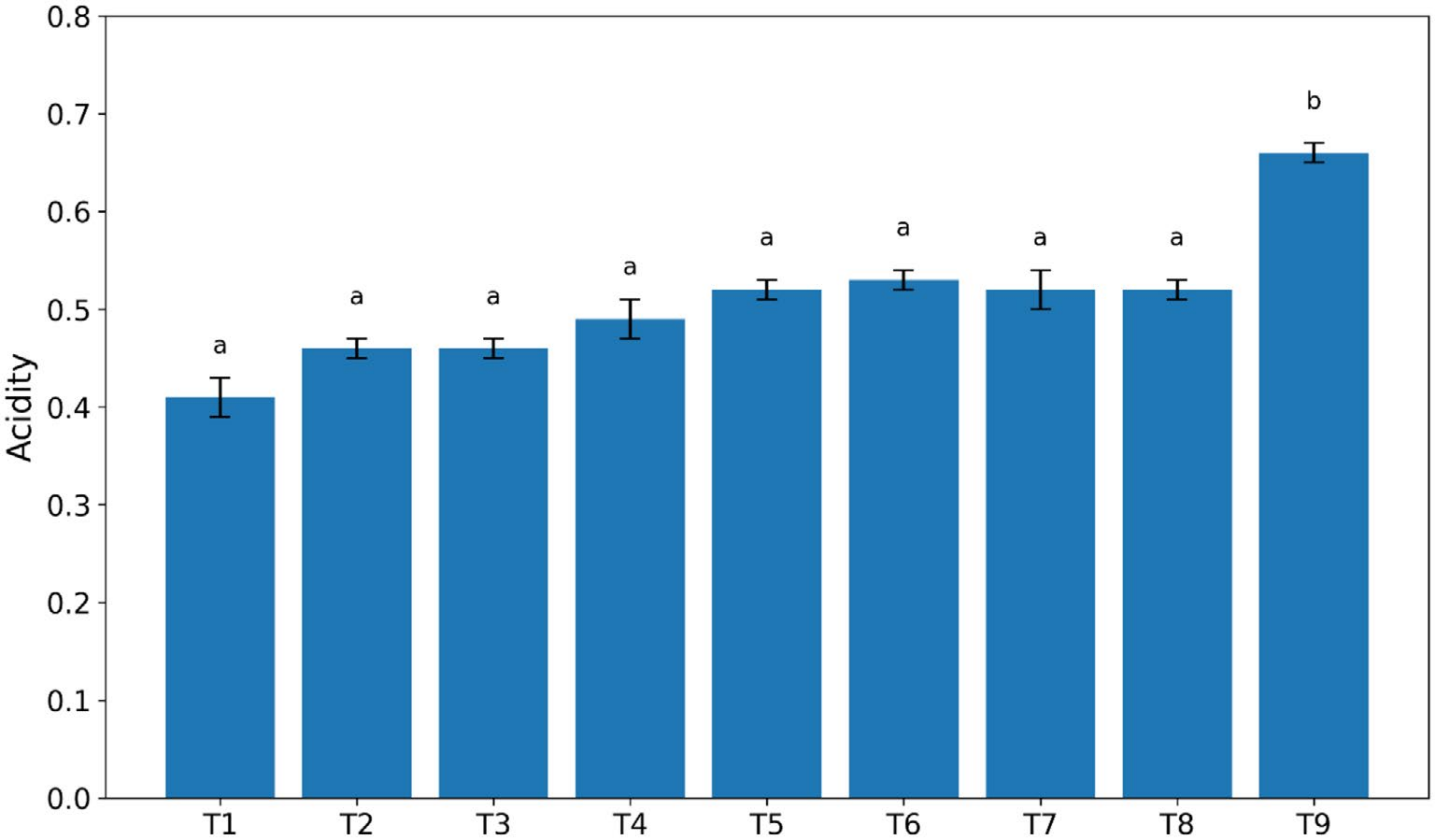
Effect of guar and Arabic gum levels on the average acidity of kiwi sauce (*n* = 3), Bars represent mean ± SD. Different letters on the bars indicate significant differences among samples (*p* < 0.05).

The acidity of different kiwi‐based sauce samples as a function of gum and stevia levels is presented in Figure 2. As shown, except for treatment number 9 (unpasteurized kiwi sauce containing stevia), the acidity of the studied samples did not differ significantly with the use of Arabic and guar gums (*p* > 0.05). In fact, there was no significant difference in acidity among the samples. The highest acidity was observed in the unpasteurized kiwi sauce containing stevia (*p* < 0.05). Additionally, the results of the acidity evaluation of the different samples showed a reverse but logical trend compared to the pH values.

### 3.3 Moisture

**FIGURE 3:**
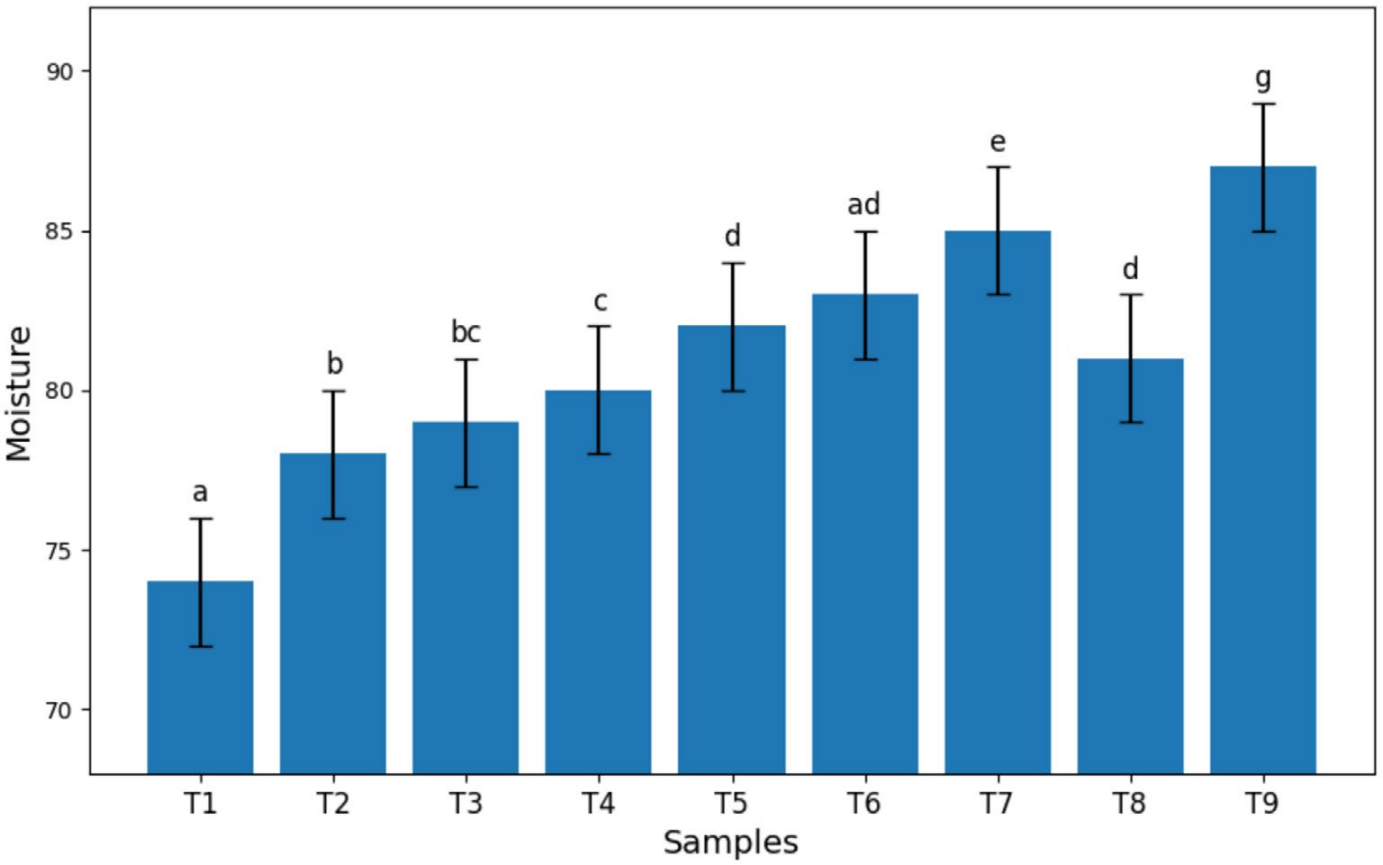
Effect of guar and Arabic gum levels on the average moisture of kiwi sauce (*n* = 3), Bars represent mean ± SD. Different letters on the bars indicate significant differences among samples (*p* < 0.05).

The effect of Arabic and guar gum levels, as well as stevia, on the moisture content of kiwi sauce is presented in Figure 3. As observed, the moisture content increased significantly with increasing guar gum percentage (*p* < 0.05), and this trend decreased with increasing levels of Arabic gum (*p* < 0.05). The highest moisture content was observed in the stevia sample.

### 3.4 Dry Matter

**FIGURE 4:**
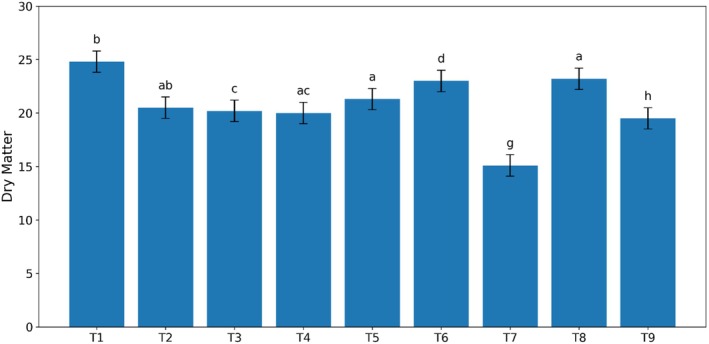
Effect of guar and Arabic gum levels on the average dry matter of kiwi sauce (*n* = 3), Bars represent mean ± SD. Different letters on the bars indicate significant differences among samples (*p* < 0.05).

The results of dry matter measurements for different kiwi sauce treatments are shown in Figure 4. As observed, the dry matter decreased significantly with increasing guar gum percentage (*p* < 0.05), while it increased significantly with increasing Arabic gum percentage (*p* < 0.05). The lowest dry matter content was observed in the sample containing stevia (15.38), which is consistent with the moisture content results.

### 3.5 Soluble Solids in Water

The percentage of soluble solids (Brix) in the produced sauces with different levels of gum and stevia is presented in Figure 4. The results showed that the content of soluble solids in the solution of the treatments increased linearly with the increase in the levels of both Arabic and guar gums used in the preparation of kiwi sauce. Among the tested samples, the lowest amount of soluble solids was observed in the sample containing stevia, and the highest amount of soluble solids was observed in the sauces containing 0.4% guar gum and also in the sauce sample containing unpasteurized sugar. This can be attributed to the presence of gum and sugar (sucrose).

### 3.6 Syneresis

The degree of syneresis of the samples was measured at 24°C, and it was only observed in the treatment containing Arabic guar. In the statistical analysis, the measured values, except for treatments 2, 3, and 9, were significant in other treatments. Syneresis is a very important factor in sauces and usually occurs due to increased molecular interactions between chains and the expulsion of water from the structure. Syneresis or water separation is an important parameter in evaluating the stability of food products during storage and is a significant challenge in processed foods. Based on the results, there is a linear and inverse relationship between the phenomenon of syneresis and the dry matter content of the product, such that groups containing Arabic gum, with lower dry matter compared to groups containing guar gum, exhibited a higher degree of syneresis. This can be explained by the possibility that Arabic gum alone is unable to prevent the separation of serum from the kiwi sauce and may only prevent syneresis when combined with other gums. The reason for this may be related to the synergistic effect with other gums in combination with each other and an increase in viscosity, which reduces syneresis and increases water absorption by the samples.

### 3.7 Colorimetry

The results of the statistical analysis of the data obtained from the color evaluation of the kiwi sauce are presented in Table 4. As can be seen, there was no significant difference between the treatments in terms of color changes (*p* > 0.05), although the color changes became more severe with increasing amounts of Arabic and guar gums, and the samples prepared with 0.4% of each gum had the most color changes compared to other samples. In fact, with increasing amounts of both guar and Arabic gums, the lightness (L component) decreased (in other words, the samples became darker) and the redness (a component) increased. The yellowness (b component) increased with increasing guar gum at the level of 0.3%, but decreased with further increases. Also, when comparing the above treatments with other tested samples, namely treatments T7, T8, and T9, no significant difference was observed in terms of color changes (*p* > 0.05).

**TABLE 4:** Color parameters L*, a*, and b* of kiwi sauce samples.

Treatments	L*	a*	b*
Kiwi sauce containing 0.2% guar gum (T1)	77.5 ± 3.1^b^	4.4 ± 3.1^a^	4.5 ± 38.8^c^
Kiwi sauce containing 0.3% guar gum (T2)	77.4 ± 1.2^b^	4.5 ± 1.1^a^	4.5 ± 35.8^c^
Kiwi sauce containing 0.4% guar gum (T3)	78.8 ± 1.5^b^	4.8 ± 1.8^a^	1.4 ± 36.5^c^
Kiwi sauce containing 0.2% Arabic gum (T4)	79.3 ± 0.8^b^	4.1 ± 3.2^a^	4.3 ± 34.5^c^
Kiwi sauce containing 0.2% Arabic gum (T5)	78.1 ± 2.5^b^	4.5 ± 1.3^a^	5.1 ± 35.1^c^
Kiwi sauce containing 0.2% Arabic gum (T6)	77.7 ± 1.1^b^	4.7 ± 5.7^a^	2.7 ± 34.2^c^
Kiwi sauce containing stevia (T7)	76.9 ± 1.8^b^	4.2 ± 4.2^a^	1.4 ± 35.4^c^
Unpasteurized kiwi sauce with sugar (T8)	77.1 ± 2.2^b^	4.4 ± 2.9^a^	2.4 ± 36.8^c^
Unpasteurized kiwi sauce with stevia sweetener (T9)	76.5 ± 1.4^b^	4.3 ± 2.4^a^	2.1 ± 34.8^c^

*Note:* Different lowercase letters between rows indicate significant differences between samples (*p* < 0/05). The data are presented as mean ± standard deviation.

### 3.8 Mold and Yeast Test

Results of the study investigating the effects of different levels of guar and Arabic gums, as well as stevia content, on mold and yeast growth in kiwi sauces revealed that although there was a significant difference in mold and yeast growth among the treatments (*p* < 0.05), all samples except for the unpasteurized sugar and stevia samples had mold and yeast counts below the standard limit of 10^2^ (see Table 5). It is worth noting that due to the role of gums in retaining moisture, they consequently decrease water activity and thus ensure high microbial stability in the product. This finding is also consistent with the results obtained from the microbial testing of the samples.

**TABLE 5:** The effect of different levels of guar and Arabic gums, and stevia on mold and yeast growth.

Treatment	T1	T2	T3	T4	T5	T6	T7	T8	T9
Test result	Negative	Negative	Negative	1 × 10^2^	2 × 10^2^	Negative	Negative	3 × 10^2^	6 × 10^2^

### 3.9 Sensory Evaluation

A sensory evaluation using a multiple comparison test was conducted by a panel of ten panelists. The evaluation was performed for the parameters of aroma and flavor, texture and consistency, taste, visual appearance, and overall acceptance. The sensory test was conducted on a 5‐point scale, with the best sample receiving a score of 5 and the worst sample receiving a score of 1. The tables below present the results of the sensory evaluation of the kiwi sauce prepared with different levels of guar and Arabic gums, as well as stevia.

Table 6 presents the results of the sensory evaluation of the 9 test samples produced with different formulations. Based on the results of the sensory evaluation, treatment number 3, that is, the sample containing 0.4% guar gum, was selected. As can be seen, the use of 0.4% guar gum significantly affected (*p* < 0.05) the sensory attributes of the kiwi sauce compared to other samples. There was no statistically significant difference between the mean scores of the 0.2% and 0.3% guar gum levels, as well as treatments 4, 5, and 6. Overall, it can be said that the sample containing 0.4% guar gum received the highest score among the sensory evaluators.

**TABLE 6:** Results of sensory evaluation.

Treatments	Aroma and flavor	Texture and consistency	Taste	Appearance	Overall acceptance
T1	1.7 ± 0.48ᵇ	1.6 ± 0.52ᵇ	1.3 ± 0.48ᶜ	1.6 ± 0.52ᶜ	2.6 ± 0.69ᵇ
T2	2.9 ± 0.57ᵇ	2.9 ± 0.57ᵇ	2.8 ± 0.63ᵇ	2.8 ± 0.63ᵇ	2.9 ± 0.57ᵇ
T3	4.6 ± 0.51ᵃ	3.8 ± 0.42ᵃ	3.6 ± 0.52ᵃ	3.8 ± 0.42ᵃ	4.3 ± 0.67ᵃ
T4	2.7 ± 0.82ᵇ	2.6 ± 0.69ᵇ	2.7 ± 0.48ᵃ	2.6 ± 0.52ᵇ	2.4 ± 0.52ᵇ
T5	2.9 ± 0.79ᵇ	2.5 ± 0.53ᵇ	1.2 ± 0.42ᵇ	2.3 ± 0.67ᵇ	2.2 ± 0.63ᵇ
T6	1.7 ± 0.48ᵇ	1.6 ± 0.52ᵇ	1.3 ± 0.48ᶜ	1.6 ± 0.52ᶜ	1.7 ± 0.48ᶜ
T7	3.9 ± 0.32ᵇ	2.7 ± 0.48ᶜ	3.7 ± 0.48ᵇ	3.1 ± 0.32ᵃ	3.8 ± 0.42ᵃ
T8	3.8 ± 0.42ᵇ	3.8 ± 0.42ᵃ	3.7 ± 0.48ᵃ	3.6 ± 0.52ᵃ	3.6 ± 0.52ᵃ
T9	3.2 ± 0.42ᵇ	3.2 ± 0.42ᵇ	3.0 ± 0.47ᵇ	2.8 ± 0.42ᵇ	2.8 ± 0.42ᵇ

*Note:* Different lowercase letters between rows indicate significant differences between samples (*p* < 0/05). The data are presented as mean ± standard deviation.

### 3.10 Rheological Analysis

The rheograms obtained for the kiwi sauce samples are presented in Figure 7. As can be seen, the viscosity index changed over the test time in treatments containing different levels of guar gum. For samples prepared with guar gum, the sample containing 0.4% guar gum showed the highest viscosity, while the sample containing 0.2% guar gum showed the lowest viscosity. The viscosity of sauces is basically attributed to their hydrocolloids.

An analysis of the given viscosity data for T7, T8, and T9 shown in Figure 9 reveals that all measurements of viscosity for T7 are far higher than those of T8 and T9 at all time periods considered, thus T7 contains a considerably more dense or viscous fluid. While T8 and T9 show relatively close levels of viscosity, T8 depicts some slight stability in viscosity over the period considered as opposed to T9, which decreases progressively with time. These results indicate that, for T7, the viscosity is high and remains alike, whereas for both T8 and T9, it is low with minimal variation. The persistence of relatively large variations in the viscosity readings between T7, T8, and T9 may be indicative of variable makeup or processing in their respective test groups; this would impact the nature of their flows.

## 4 Discussion

Hydrocolloids are commonly used to improve the properties, create new textures, and characteristics in textured products, as well as to reduce production costs in food. The selection of hydrocolloids is based on the desired functional properties in the final product. In the first phase of this research, guar and Arabic gums were used at three levels of 0.2%, 0.3%, and 0.4%, and the optimal gum percentage for kiwi sauce preparation was determined. In the second phase, the natural sweetener stevia was used as a sugar substitute in the routine sauce formulation. Finally, pasteurized and unpasteurized kiwi sauce products containing sugar and stevia, along with other prepared treatments, were compared based on physicochemical tests and sensory evaluation, and the best sample was selected. In the following, we will analyze the findings of this research.

Based on the conducted experiments, the effect of different levels of guar and Arabic gums, as well as stevia, on the pH and acidity of kiwi sauce was evaluated (Figure 1). The results of the analysis of variance showed no significant difference between the experimental treatments, and consequently, no significant change in acidity was observed. These results are consistent with the reports of Rezaei et al. (2011) who stated that the use of hydrocolloids or stabilizers has no significant effect on the pH of frozen yogurt samples. In contrast, Ardali et al. (2014) reported a decrease in pH in orange juice samples with increasing percentages of stevia. However, Alizadeh et al. (2014), who investigated the effect of replacing sugar with stevia in the production of fruit‐flavored milk drinks, concluded that the type of sugar had no effect on the pH of the samples. It should be noted that the difference in pH changes of a food product in the presence of stevia probably depends on the type, food system, and other soluble compounds present in it.

The results of the moisture test (Figure 3) showed that the highest moisture percentage was attributed to the stevia sample. This can be attributed to the presence of stevia alcohol in the kiwi sauce formulation, as sugar alcohols tend to retain water in their structure due to the presence of hydroxyl groups (Akesowan 2009). These results are similar to the findings of Ronda et al. (2005), and Jali et al. (2013). In addition to the hydroxyl groups present in stevia alcohol, the increase in moisture content in samples containing stevia can be attributed to the effect of the protein present in stevia, which creates a gel‐like network, leading to structural stability against heat and water retention in the three‐dimensional gel network, and consequently, an increase in the moisture content of the final product. In fact, one of the advantages of stevia powder is its high water‐holding capacity, which is apparently due to its high protein content (Lemus‐Mondaca et al. 2012).

On the other hand, one of the characteristics of hydrocolloids such as Arabic gum is their hydrophilicity. In fact, hydrocolloids affect the moisture content and water activity of food products by creating aqueous bonds. Also, based on the results obtained, the moisture content of the samples increased with increasing guar gum. Khazaei et al. (2014) showed that the moisture content of kiwi pastille samples increased with increasing guar gum in the formulation, which is also consistent with the results of the present study.

**FIGURE 5:**
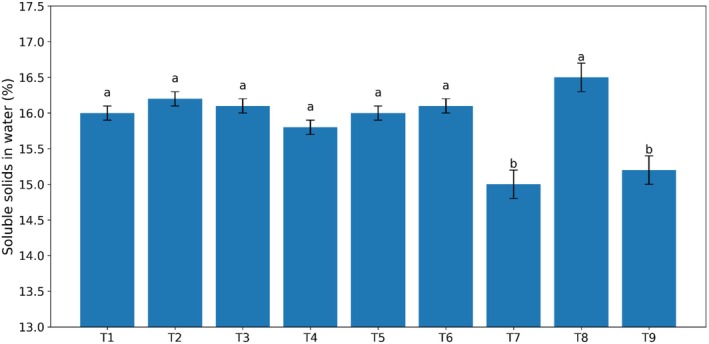
Effect of guar and Arabic gum levels on the average brix of kiwi sauce (*n* = 3), Bars represent mean ± SD. Different letters on the bars indicate significant differences among samples (*p* < 0.05).

Another parameter examined was the percentage of water‐soluble solids in the test samples (Figure 5). The results showed that the percentage of solids in the solution of treatments increased linearly with increasing levels of guar gum and Arabic gum in the preparation of kiwi sauce (*p* < 0.05). Guar gum and Arabic gum are expected to increase Brix due to their solubility in water. As observed, the lowest amount of solids in the solution belonged to the sample containing stevia, and the highest amount of solids in the solution belonged to the sauces containing 0.4% guar gum and also to the pasteurized sauce sample containing sugar. The reason for this can be attributed to the presence of sucrose and gums used in the kiwi sauce formulation; thus, increasing the amount of sucrose increases the percentage of soluble solids. The results of this research were consistent with the results of (Alizadeh et al. 2014). Also, in another study conducted by Razavi et al. (2014), the results showed that the percentage of water‐soluble solids (Brix) increased; they attributed this to the presence of soluble substances, especially sugars such as glucose, galactose, arabinose, etc., in the structure of these types of gums.

**FIGURE 6:**
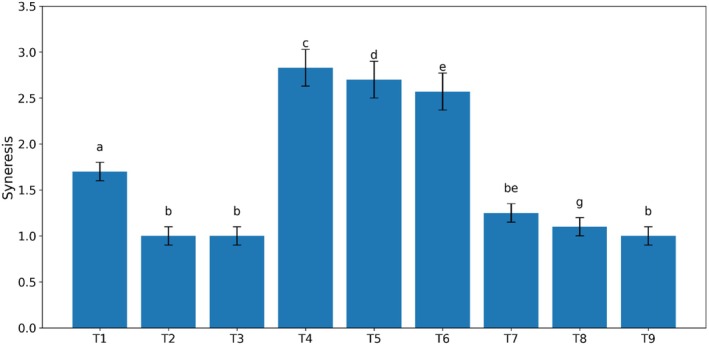
Effect of guar and Arabic gum levels on the average syneresis of kiwi sauce (*n* = 3), Bars represent mean ± SD. Different letters on the bars indicate significant differences among samples (*p* < 0.05).

Another parameter examined was the phenomenon of syneresis or water separation (Figure 6). The test results showed that it was negative. Based on the results obtained, there was a linear and inverse relationship between syneresis and the product's dry matter content, so that groups containing Arabic gum, due to having less dry matter compared to groups containing guar gum, had a higher degree of syneresis. In fact, the absence of syneresis means the ability to retain water within the sauce tissue, and this feature, in addition to maintaining the appearance of the product's texture, is also effective in shelf‐life and preventing the growth of microorganisms (Aleman et al. 2011). Syneresis is a very important factor in sauces and is usually caused by an increase in molecular bonds between chains and the release of water from the structure. Syneresis or water loss is an important parameter in evaluating the stability of food products during storage, which is a significant challenge in processed foods and controlling it has high commercial importance. The addition of different hydrocolloids, due to their importance in water solubility and high molecular weight, improves the textural and rheological properties of the product and is used to increase viscosity, create a gel‐like structure, and reduce syneresis or water separation (Aliakbari and Mansouri‐Pour 2017; Raftani et al. 2016; Shakiba et al. 2017). Omidebakhsh et al. (2012) reported that with increasing gum concentration in the test treatments, syneresis decreased, which was attributed to the high water‐holding capacity of the gum.

Among the physical properties of food, color is recognized as the most important visual attribute in the perception of quality (Table 3). Customers tend to associate color with the flavor, safety, shelf life, quality, and nutritional properties of food products (Pedreschi et al. 2006). As the results presented, with increasing the percentage of gum in the samples, the amount of b*, that is, yellowness of the product, decreased approximately, which was not statistically significant (*p* < 0.05). Also, the amount of a* and L* did not have a significant difference between the different treatments and no specific trend was observed. Rafie and Qiafeh Davoodi (2021) reported that adding the gums used increased the amount of L* and a* components. However, adding these compounds did not have a significant effect on the amount of b* components. It seems that the reason for this is the high ability of guar gum and Arabic gum to retain moisture.

In the microbial analysis of the studied samples, the results of investigating the effect of different levels of guar and Arabic gums, as well as stevia on the total bacterial count and the growth of mold and yeast in the prepared sauces are presented in Table 4. It is indicated that none of the bacteria, molds, and yeasts showed significant growth, and the results of the mold and yeast microbial tests were in accordance with the national Iranian standard No. 2965 related to the microbiological characteristics of kiwi sauce. The results of the research by Aghaee et al. (2013) showed that stevioside has antimicrobial effects on food spoilage bacteria, which confirms the findings of the present study. According to the statistical analysis obtained, in all cases, there was a direct relationship between bacterial growth and the amount of stevia. This trend of effect on bacterial growth indicates that the extract of this plant has a specific antibacterial effect that increases with increasing concentration or, in other words, with increasing the active ingredient. Fazel et al. investigated the antimicrobial effects of stevia on the growth of spoilage‐causing bacteria *Bacillus subtilis* , *Staphylococcus aureus* , *Escherichia coli* , *Pseudomonas aeruginosa* , and *Klebsiella pneumoniae* . The results of their research showed the antimicrobial effect of stevia on these species, which is consistent with the results of the present study (Fazal et al. 2011). In fact, the antimicrobial effect of stevia can be attributed to the presence of various types of flavonoids, alkaloids, steroids, tannins, and terpenes (Sato et al. 1996).

**FIGURE 7:**
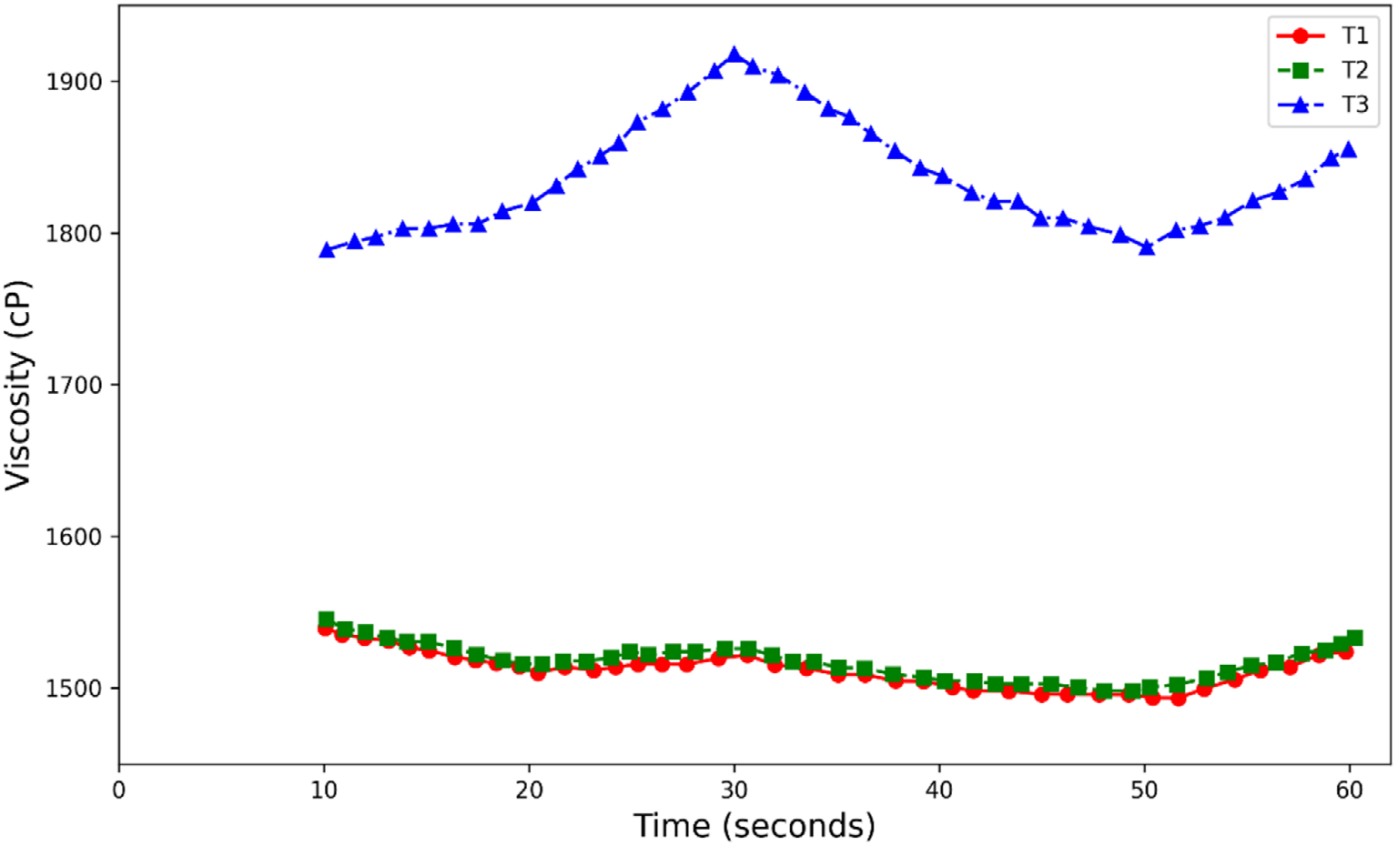
Viscosity variation in terms of time in treatments containing different levels of guar gum.

Viscosity in a sauce is a physical property directly linked to its quality and perceived mouthfeel. Generally, an increase in viscosity in samples containing gum (Figure 7) can be attributed to the gum's interaction with the liquid portion of the mixture and its high water‐binding capacity. These compounds, by increasing their capacity to bind water, decrease flowability and increase the sample's resistance to flow, or in other words, its viscosity (Rezai et al. 2010). Furthermore, increasing the gum concentration can lead to the formation of stronger networks and structures in the presence of the gum (Omidebakhsh et al. 2012). Aminigo et al. (2009) reported that adding hydrocolloids increases water‐binding capacity, leading to two significant physical effects: a decrease in syneresis and an increase in apparent viscosity. Tamime et al. (2007) reported that hydrocolloids increase viscosity by binding free water in the sample, which is consistent with the results of this study.

**FIGURE 8:**
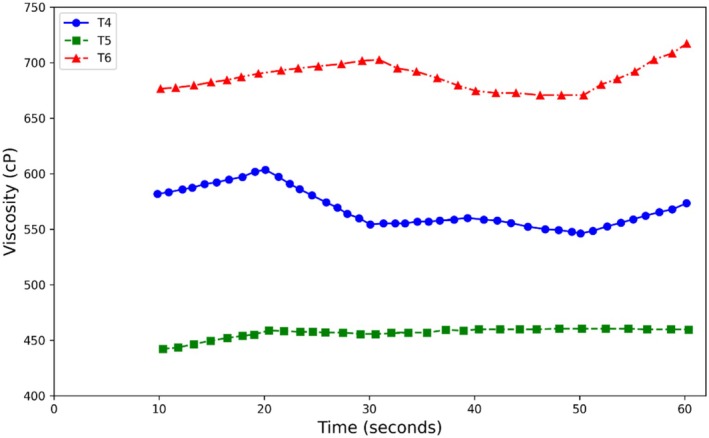
Viscosity variation in terms of time in treatments containing different levels of Arabic gum.

As expected, at equal concentrations, guar gum caused a greater increase in viscosity compared to Arabic gum (see Figures 7 and 8). This increase in viscosity at low concentrations is due to the ease with which water is absorbed. Guar gum and Arabic gum are classified as dietary fibers and are expected to exhibit the physicochemical properties of fibers. They increase viscosity due to an increase in serum concentration as a result of water retention by the fibers (Rezai et al. 2010).

**FIGURE 9:**
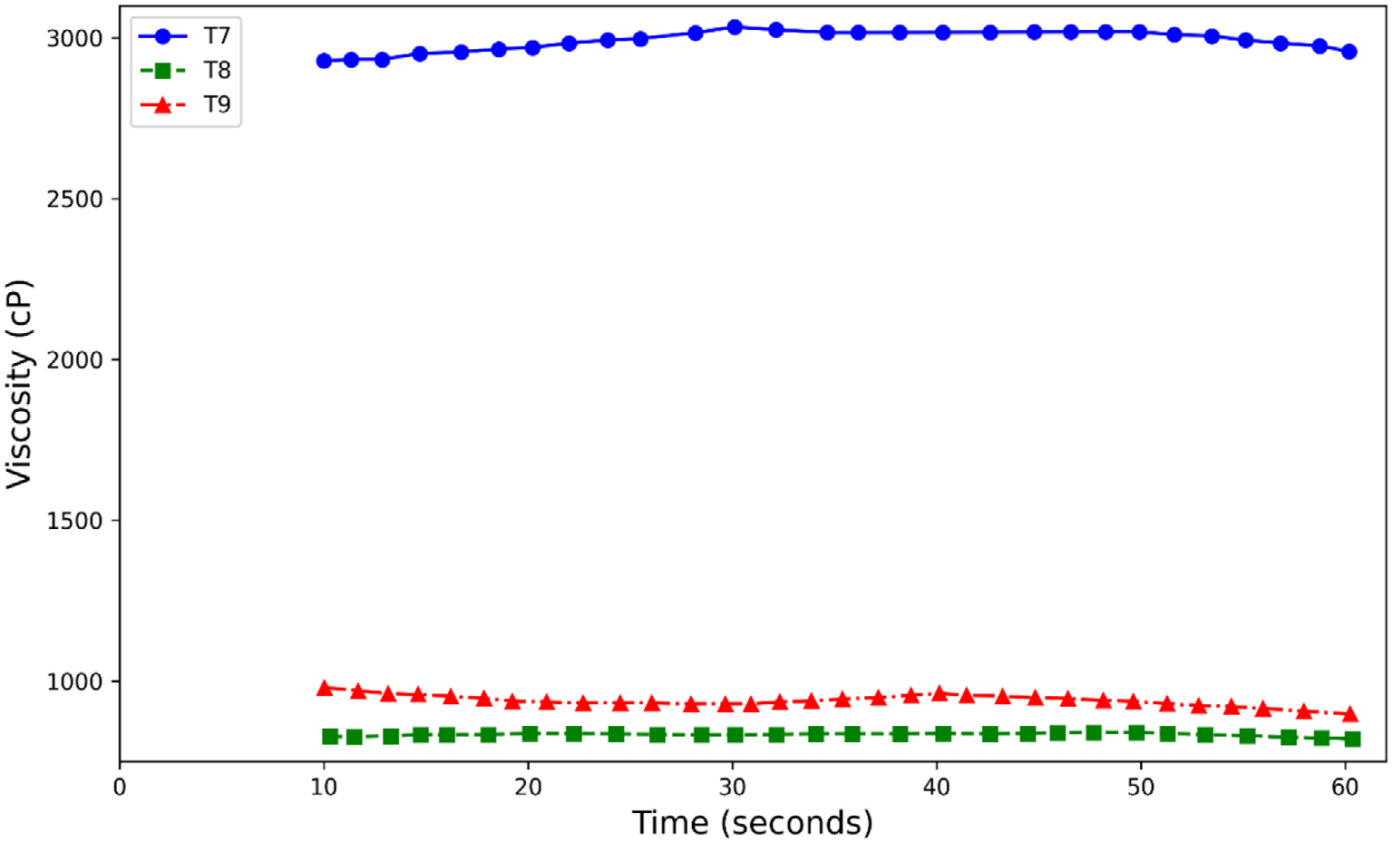
Viscosity variation in terms of time in treatments containing stevia, unpasteurized stevia, and unpasteurized sugar.

Furthermore, a comparison of the viscosity of samples containing stevia and sugar (sucrose) revealed that samples containing stevia exhibited greater changes in viscosity (Figure 9). This can be explained by the fact that most sugars, due to their strong hydrophilic properties and solubility, produce concentrated solutions. Sugars form hydrogen bonds with water molecules through their hydroxyl groups, leading to an increase in viscosity (Fennema 1996). Considering the chemical structure of stevia sweetener and its higher number of free functional groups compared to sucrose, hydrogen bonds are increased, resulting in a decrease in free water mobility and, consequently, an increase in the viscosity of kiwi sauce. The results of the research by Guggisberg et al. (2011) on the increase in the viscosity of yogurt containing stevia, as well as Lisak et al. (2011) on the increase in the viscosity of strawberry yogurt with the addition of stevia, are consistent with the results of this study. On the other hand, natural sweeteners are hygroscopic and their tendency to absorb water increases viscosity (Kashiri et al. 2009). The intensity of the sweetener's tendency to absorb water depends on its size and molecular weight. The lower the molecular weight of sugars, the greater the tendency to absorb water and the higher the viscosity. Sugar alcohols increase viscosity in products. Additionally, molecular chains increase hydrogen bonding and increase intermolecular connections, leading to increased viscosity (Chole 2000).

## 5 Conclusion

This research investigated the effects of adding different amounts of guar gum, Arabic gum, and stevia on the physicochemical, rheological, and sensory properties of kiwi sauce. The results showed that adding hydrocolloids, particularly at higher concentrations, significantly impacted the sauce's moisture content, dry matter, and syneresis (liquid separation), leading to improved stability and texture. Guar gum at a concentration of 0.4% was most effective in increasing viscosity, creating a desirable thick consistency for fruit sauces. Stevia, used as a natural sweetener, provided a healthier sugar alternative while maintaining acceptable taste and microbial stability. This study demonstrates that incorporating hydrocolloids and natural sweeteners can improve sauce quality, leading to healthier and more stable fruit‐based products. Future research should explore consumer acceptance and long‐term storage stability to further evaluate commercial viability.

## Author Contributions

**Seyede Golsa Mousavi:** data curation (equal), investigation (equal), methodology (equal), validation (equal), visualization (equal), writing – original draft (equal). **Leyla Alizadeh:** conceptualization (equal), formal analysis (equal), supervision (equal), writing – review and editing (equal). **Mostafa Karami:** methodology (equal), software (equal), writing – review and editing (equal).

## Conflicts of Interest

The authors declare no conflicts of interest.

## Data Availability

The datasets produced and/or analyzed in this study are accessible upon reasonable request from the corresponding author.
